# Recurrent *SETD2* mutation in *NPM1*-mutated acute myeloid leukemia

**DOI:** 10.1186/s40364-020-00243-y

**Published:** 2020-11-11

**Authors:** Jiewen Sun, Wenjuan Yu, Xiang Zhang

**Affiliations:** 1 Center Laboratory, Affiliated Secondary Hospital, Zhejiang Chinese Medical University, Zhejiang, Hangzhou China; 2 Department of Hematology, The First Affiliated Hospital, Zhejiang University School of Medicine, #79 Qingchun Rd, Zhejiang, 310003 Hangzhou China; 3 Key Laboratory of Hematologic Malignancies, Diagnosis and Treatment, Zhejiang, Zhejiang, Hangzhou China

**Keywords:** *SETD2* mutation, *NPM1* mutation, Acute myeloid leukemia

## Abstract

SETD2 is the only methyltransferase for H3K36me3, and our previous study has firstly demonstrated that it functioned as one tumor suppressor in hematopoiesis. Consistent with it, *SETD2* mutation, which led to its loss of function, was identified in AML. However, the distribution and function of *SETD2* mutation in AML remained largely unknown. Herein, we integrated *SETD2*-mutated AML cases from our center and literature reports, and found that *NPM1* mutation was the most common concomitant genetic alteration with *SETD2* mutation in AML, with its frequency even higher than *MLL* rearrangement and *AML1-ETO*. Though this result indicated the cooperation of *SETD2* and *NPM1* mutations in leukemogenesis, our functional study showed that *SETD2* was required for the proliferation of *NPM1*-mutated AML cell line OCI-AML3, but not *MLL*-rearranged AML cell line THP-1, via maintaining its direct target *NPM1* expression, which was just opposite to its role of tumor suppressor. Therefore, we speculated that SETD2 possibly had two different faces in distinct subtypes and stages of AML.

**Supplementary Information:**

The online version contains supplementary material available at 10.1186/s40364-020-00243-y.

**To the editor**

SETD2 has been demonstrated as one tumor suppresser in hematopoiesis [1], and *SETD2* mutation affected AML, in which its distribution remained not fully understood [2]. Herein, we analyzed the *SETD2* mutation in *NPM1*-mutated AML.

One 36-year-old woman was committed due to abdominal pain and fever for 7 and 3 days, respectively. PB test showed WBC: 52.4 × 10^9^/L, Hb: 98 g/L, PLT: 48 × 10^9^/L, circulated blast: 80%. BM examination exhibited 67.5% myeblasts with the immunophenotype of CD11b-CD13^dim^ + CD14-CD15^dim^ + CD33 + CD34^partial^ + CD35-CD38^dim^ + CD45 + CD64-CD65^dim^ + CD71 + CD117 + CD123^dim^ + HLA-DR-. Though karyotype was normal and *CBF* or *MLL* rearrangements were negative, *NPM1*, *SETD2*, *NRAS* and *ETV6* mutations were identified. Therefore, AML with mutated *NPM1* was diagnosed. After receiving the operation for co-existed acute appendicitis, she accepted IA regimen as induction therapy, and CR1 was achieved. Subsequently, she received three cycle of medium-dose cytarabine regimen. However, AML relapsed at the 3 months after cessation of chemotherapy, and 72% myeloblasts re-emerged in BM. Due to the early recurrence, she accepted HAA and CLAG regimen successively, and achieved CR2. However, the leukemic clones were not eradicated reflected by persistent above mutations. Therefore, allogeneic semi-compatible HSCT was immediately conducted. As follow-up, CR was still maintained at the 15 months after HSCT **(**Fig. 1a).

**Fig. 1:**
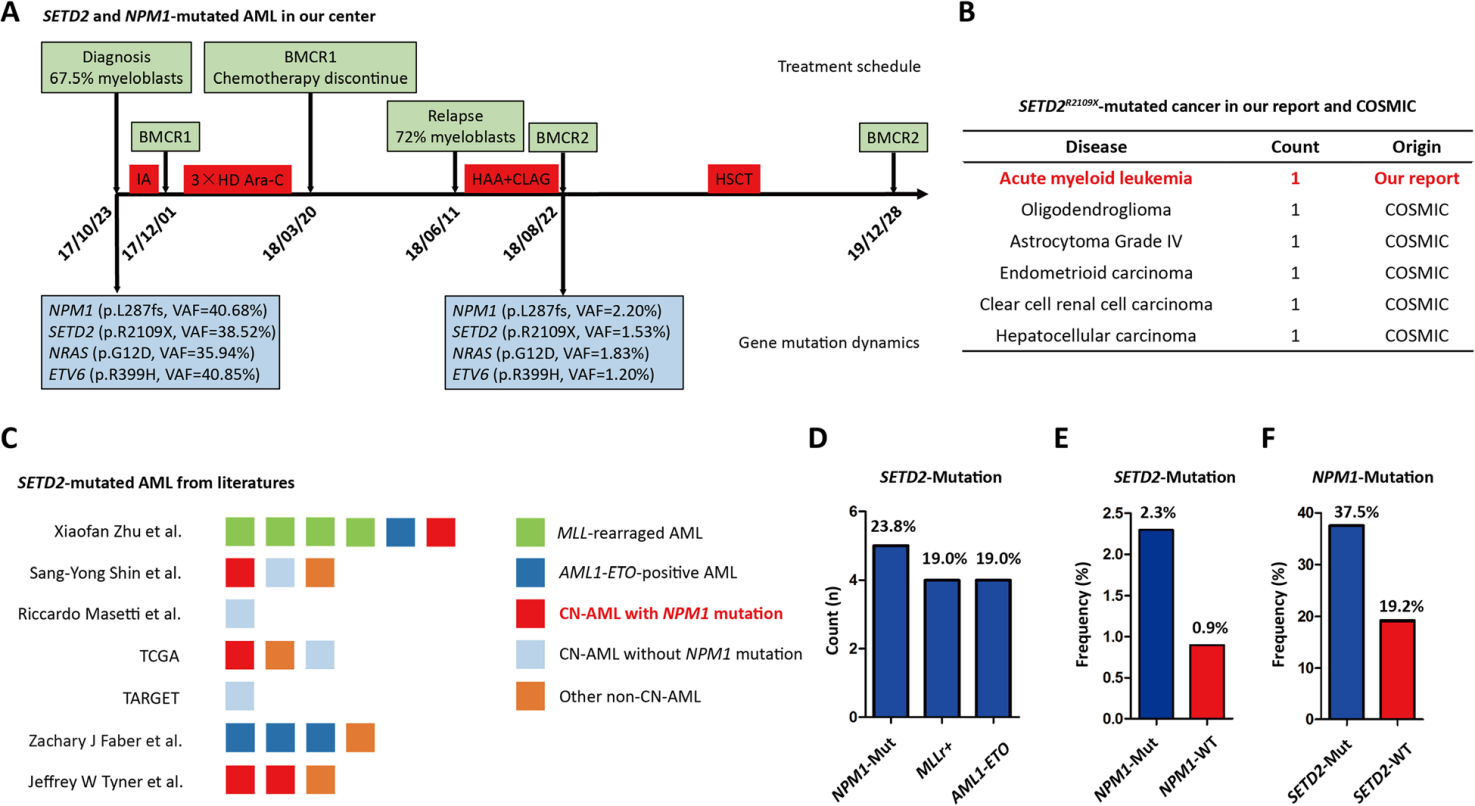
The distribution of *SETD2* mutation in AML. **a** One *SETD2*-mutated AML case in our center. **b** *SETD2*^*R2109X*^-mutated cancers in our report and COSMIC database. **c** *SETD2*-mutated AML cases from literature reports. **d** The common concomitant genetic alterations with *SETD2* mutation in AML patients from literature reports. **e** and **f** The frequency of *SETD2* mutation in *NPM1*^*Mut*^ or *NPM1*^*WT*^ AML (**e**) and it of *NPM1* mutation in *SETD2*^*Mut*^ or *SETD2*^*WT*^ AML (**f**) were calculated, and all AML cases from our study and literature reports mentioned above were involved

In this patient, *SETD2*^*R2109X*^ was identified, and it was also found in other malignancies from COSMIC database **(**Fig. 1b**)**, so *SETD2*^*R2109X*^ was one driver in cancer. However, *SETD2* deficiency was not sufficient to generate AML, so additional hits were required [1, 3]. Therefore, we reviewed AML studies involving *SETD2* mutation [2, 4–9], and found that *NPM1* mutation rather than *MLL* rearrangement or *AML1-ETO* was the most common co-existed genetic alteration of *SETD2* mutation in AML **(**Fig. 1c-d). To establish their association, we displayed subgroup analysis in above studies, then submitted it Pearson’s chi-square test, and calculated OR. Strikingly, *SETD2* and *NPM1* mutations were the concomitant mutation in AML (*P* = 0.031; OR = 3.28) **(**Fig. 1e-f). To address whether *SETD2* mutation mediated drug resistance in AML, we analyzed their therapeutic response to standard chemotherapy. Among 22 *SETD2*-mutated AML patients, the data were available in 11 patients, while CR, PR, and NR was 72.7%, 9.09%, and 18.2%, respectively. Notably, the CR was comparable to it in total AML. Interestingly, all with *NPM1*-mutated AML achieved CR, and two with *MLL*-rearranged AML exhibited NR. Therefore, *SETD2* mutation was possibly not one determinant in drug sensitivity for AML. Furthermore, we analyzed the OS between *SETD2*- mutated and wild-type groups with cBioPortal database [10, 11], but no significance between two groups was found (Additional file 1: Figure S1). Regretfully, the data about EFS were not available.

Loss of SETD2 function accelerated the progression of *MLL*-rearranged or *AML1-ETO*-positve AML, but whether it was the same in *NPM1*-mutated AML remained unknown. Herein, we displayed shRNA-mediated *SETD2* knockdown, which simulated its loss of function caused by *SETD2* frame-shift or nonsense mutation, in *NPM1*-mutated AML cell line OCI-AML3 and *MLL*-rearranged AML cell line THP-1. Interestingly, *SETD2* knockdown impaired the proliferation of OCI-AML3 but not THP-1 cells **(**Fig. 2a-d). Furthermore, the proliferative defect of OCI-AML3 was caused by increased cell apoptosis **(**Fig. 2e) and cell cycle arrested at G1/G0 phase **(**Fig. 2f). It has been reported that the viability of OCI-AML3 relied on the function of *NPM1* mutation [12], while *NPM1* expression was regulated by the transcriptional activation mark, H3K36me3, which indicated by ChIP-Seq in the HSPCs of *Mll-af9*-positive leukemia **(**Fig. 2g) [13]. Consistently, we demonstrated that *NPM1* and its direct targets *MEIS*, *HOXA9* were significantly down-regulated in *SETD2* knockdown OCI-AML3 cells **(**Fig. 2h-i**)**. Therefore, our results indicated that *SETD2* knockdown-mediated OCI-AML3 proliferation inhibition was possibly attributed to *NPM1* down-regulation.

**Fig. 2:**
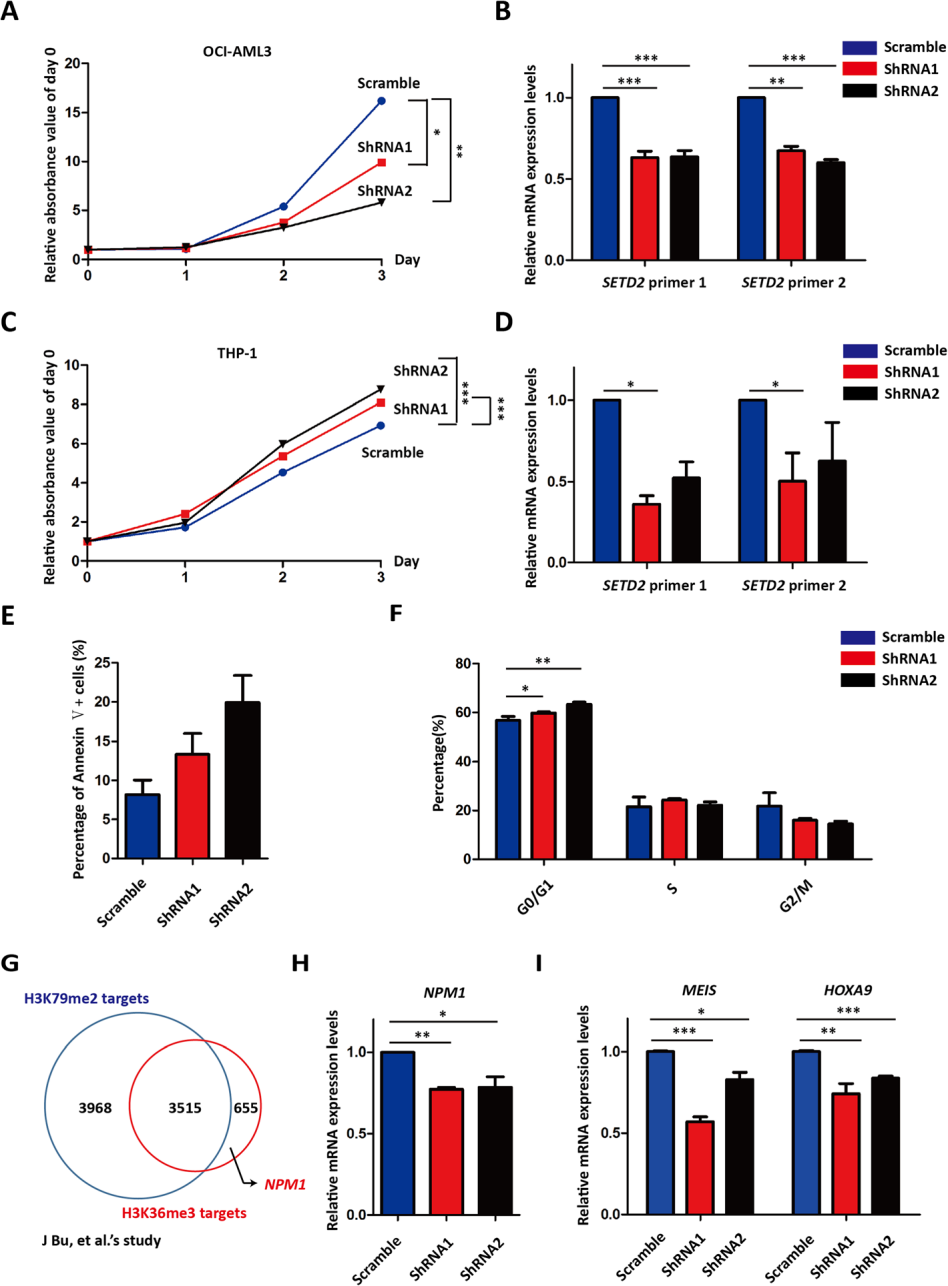
*SETD2* was required for the maintenance of *NPM1*-mutated AML cell line OCI-AML3. **a** and **b** The proliferation (**a**) and *SETD2* expression (**b**) of *scramble* and *SETD2* knockdown OCI-AML3 cells. **c** and **d** The proliferation (**c**) and *SETD2* expression (**d**) of *scramble* and *SETD2* knockdown THP-1 cells. **e** Annexin-V staining for detecting cell apoptosis in OCI-AML3 cells. **f** PI staining for cell cycle analysis in OCI-AML3 cells. **g** *NPM1* has been demonstrated as one direct target of H3K36me3 in the literature report. **h** and **i** The expression of *NPM1* (**h**) and its direct downstream targets, *MESI* and *HOXA9* (**i**), was analyzed in *scramble* and *SETD2* knockdown OCI-AML3 cells. ***, *P* < 0.001; **, *P* < 0.01; *, *P* < 0.05; T test was used for each graph

The detailed role of *SETD2* mutation in *NPM1*-mutated AML remained mysterious. Theoretically, *SETD2* and *NPM1* mutations probably cooperated in leukemogenesis. However, our results showed that *SETD2* was required for the maintenance of OCI-AML3. To our knowledge, two possibilities existed: firstly, *SETD2* mutation played different roles in the initiation and maintenance of *NPM1*-mutated AML; secondly, additional genetic alteration influenced SETD2 function in *NPM1*-mutated AML. Therefore, further investigations were needed in the furture.

## Supplementary Information

**Additional file 1: Figure S1.** The OS of *SETD2*- wild type and mutated AML patients from the summary of TCGA, TARGET, and OHSU studies.

## Data Availability

All data generated or analyzed during this study are included in this published article.

## Abbreviations

AL: Acute leukemia
ALL: Acute lymphoblastic leukemia
AML: Acute myeloid leukemia
BM: Bone marrow
ChIP-Seq: Chromatin immunoprecipitation-sequencing
CLAG: Cladribine, cytarabine plus granulocyte colony-stimulating factor
CR: Complete remission
EFS: Event-free survival duration
H3K36me3: Tri- methylated histone 3 lysine 36
HAA: Homoharringtonine, aclacinomycin, plus cytarabine regimen
HB: Hemoglobin
HSCT: Hematopoietic stem cell transplantation
HSPCs: Hematopoietic stem progenitor cells
IA: Idarubicin plus cytarabine regimen
MDS: Myelodysplastic syndrome
NR: No response
OR: Odds ratio
OS: Overall survival duration
PB: Peripheral blood
PLT: Platelet
PR: Partial remission
WBC: White blood cell
