# Supplementary figures and images for: Recurrent SETD2 mutation in NPM1-mutated acute myeloid leukemia
Source: Biomark Res. 2020 Nov 11;8:62. doi: 10.1186/s40364-020-00243-y (PMC7659109; doi:10.1186/s40364-020-00243-y)

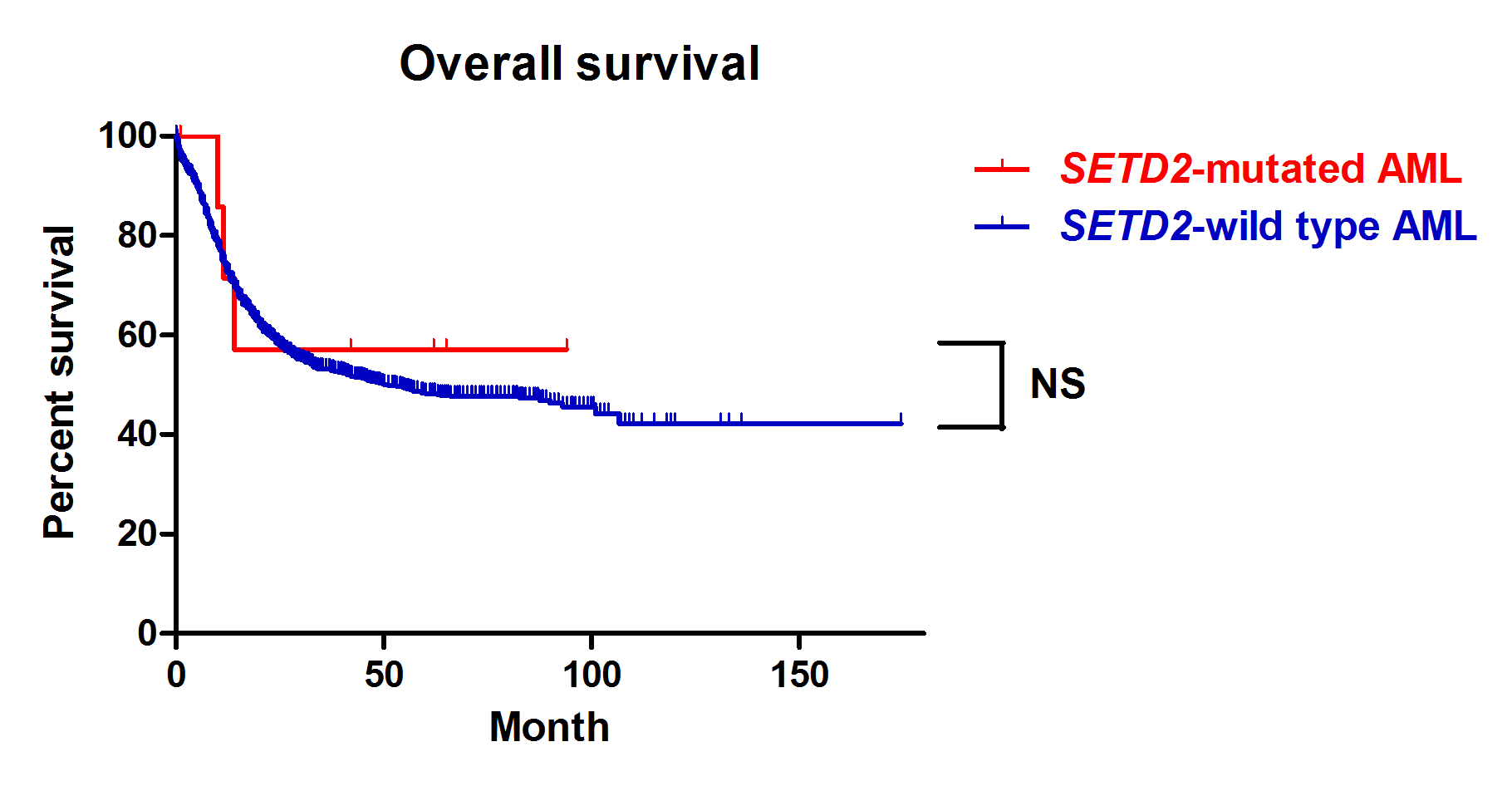

Supplement: Supplementary file 1 — Additional file 1: Figure S1. The OS of SETD2- wild type and mutated AML patients from the summary of TCGA, TARGET, and OHSU studies. [file 40364_2020_243_MOESM1_ESM.tif]
